# Do They Need Goals or Support? A Report from a Goal-Setting Intervention Using Physical Activity Monitors in Youth

**DOI:** 10.3390/ijerph13090914

**Published:** 2016-09-13

**Authors:** Michal Bronikowski, Malgorzata Bronikowska, Agata Glapa

**Affiliations:** 1 Department of Physical Activity Didactics, University School of Physical Education, Krolowej Jadwigi 27/38, 61871 Poznan, Poland; bronikowski.michal@wp.pl; 2 Department of Traditional Games and Ethnology of Sport, University School of Physical Education, Krolowej Jadwigi 27/38, 61871 Poznan, Poland; wadera43@gmail.com

**Keywords:** youth, physical activity, goal strategy, classmate support, teacher support, activity monitors

## Abstract

The objective of this study was to investigate the association between physical activity (PA) and different goal setting and strategies in youth. The study took into consideration different sources of support as well as gender variations. Classmate and Teacher Support scales were used to evaluate support in physical education (PE) classes, and moderate-to-vigorous physical activity (MVPA) was reported. Garmin Vivofit^®^ activity trackers were used during an 8 week-long intervention to count daily steps. Data was collected from 65 adolescents (mean age 17.2 ± 0.2), 74 young adolescents (mean age 15.3 ± 0.2) and 57 children (mean age 11.5 ± 0.4). An experimental design was employed, with “goal” and “do your best” groups given different step goal strategies. The results show that both groups achieved a comparable number of steps. Two-way ANOVA showed interactional effects between gender and teacher support. There were no such effects for MVPA and number of steps. Although classmate support in PE was reported to be reasonably high, the findings show that it does not play a significant role in increasing MVPA behaviors in youths. However, the problem of significantly lower support given to adolescent girls by PE teachers should be embedded into the teaching context of PE students and counteracted in school setting realities.

## 1. Introduction

PA has been related to positive outcomes in both physical and psychological terms [1], but despite this, the PA levels decline with age from late childhood and adolescence [2], with girls being less active than boys at all ages [3]. World Health Organization (WHO) recommendations for 6–18 year olds [4] on 60 min of MVPA a day—consisting of mainly aerobic activities, but with bouts of vigorous intensity and muscle strengthening exercises as well—are not met by majority of youth [5]. Findings from various research studies indicate that for adolescents 8000 to 12,500 steps per day equal the recommended level of 60 min of MVPA [6,7]. Research by Tudor-Locke et al. [8] has shown that the daily range of steps in young females is 10,000–13,000, but, as with PA levels, this steadily declines to approximately 8000–9000 steps per day in 18-year-olds. Normative data indicates that healthy adults typically take between 4000 and 18,000 steps per day, and that 10,000 steps per day is a reasonable goal target for the adolescent population [8].

In recent years there have been a number of intervention studies on PA in youth, with an increasing number of these using accelerometer- and pedometer-based activity monitors to collect multiple data measurements. Activity monitors’ visual displays provide real-time estimates of activity levels and give easy-to-follow feedback to the person engaging in a given activity. This is important when researching and meeting the challenge of changing health-related behaviors. Research on patients [9] show that feedback information enhances sustained adoption of healthy behaviors. Wearable activity trackers (usually worn as wrist bands) can be used for monitoring fitness and other health-related metrics (such as step counts and distance covered). According to Evenson, et al. [10] accelerometer step counting had a high (0.80) correlation with tracker-assessed steps (pedometers). The use of such devices providing health information can empower individuals, enable them to cope with varying situations, and make better health-related choices, which is in line with recommendations made by, for example, the Ottawa Charter for Health Promotion [11].

Monitoring goal-related progress is an important moderator of goal achievement because it provides the information necessary for adjusting strategies and/or effort levels [12,13]. Locke et al. [14] found that setting more specific and challenging (yet achievable) goals and providing feedback information on goal attainment enhance self-efficacy, in turn help to mobilize a person’s resources.

Goal-setting theory, developed by Locke and Latham [15] provides evidence of effective goal-setting strategies that have been used in intervention research to change health-related behaviors. Goal setting theory describes the relationship between goals and behavior with the focus being on a person’s choice of goals, their motivation to achieve the goals, and the likelihood that the goals will be achieved [16]. There are two major components to this strategy: the content of the goal, which is related to its specificity and difficulty; and the intensity of the goal, which is related to the effort required, the importance or priority it is given, and a person’s commitment to the goal [15]. Locke and Latham [16] proposed that as long as a person is committed to their goal, has the ability to achieve their goal, and has no opposing goals, then there is a positive linear relationship between goal difficulty and performance. Goal setting is also addressed in Social Cognitive theory, which emphasizes the importance of setting achievable goals as a way to increase self-efficacy which, subsequently, can lead to behavior change [17]. Bandura [18] argued that goal strategy may be more effective, especially in terms of self-efficacy, when individuals set specific, proximal and moderately difficult goals. However, elsewhere Stracher et al. [19] found that, when compared with a strategy of not setting any quantitative goals, such as ‘do your best’, setting specific difficult goals leads to higher performance. This has also been confirmed by other studies, although mainly in adults rather than children [11].

Deci and Ryan [20] emphasize the importance of intrinsic motivation for lifelong psychological growth, which allows people to engage in a task they find interesting and challenging. So is the case with the fundamental needs, such as competence, relatedness, and autonomy, the satisfaction of which leads to increased well-being. Understanding that autonomous and controlled tasks involve different types of regulatory processes: intentional (i.e., motivated) behavior [20] the present study developed, and evaluated utility of a school-based intervention targeted to change pupil’s PA levels.

A recent research study by Eather et al. [21] shows that social support from PE teachers influences PA levels in children, but that neither support from parents nor friends had a mediating effect on PA. Similar effects were found in adolescents by Lubans et al. [22]. However, research in this area is sparse. In another study [23], the engagement of parents together with adolescents (being active together) strongly influenced leisure-time PA, whereas engagement with other peers was related to more vigorous PA and competitive sports [24]. Dishman et al. [25] found that female adolescents with high social support generally experience lower declines in PA levels when they also have high self-efficacy. Importantly though, the study also indicated that when adolescents perceive a reduction in social support, their PA decreases, even if their self-efficacy remains high.

Studies on the role of support in PA [26] show that the effect of perceived autonomy support from peers and parents on leisure-time PA were small and inconsistent, while support from PE teachers produced a unique effect. Research findings from a number of studies have led Deci and Ryan [27] to point to the importance of autonomy-supportive teachers and classrooms, but the relation is not quite so clear cut. Mendoca et al. [28], who analyzed a number of studies, found no (or only limited) association between PA and social support from teachers. Elsewhere, Koka [29] found that although support from both PE teachers and peers had significant and positive effects on autonomous motivation to participate in PE among 12–16 years old students, it was only perceived support from the teacher that had an indirect effect on students’ leisure-time PA behavior through perceived autonomy and competence. Koka [29] concludes that although support from both PE teachers and peers are essential antecedents to the satisfaction of perceived psychological needs in PE, in reality it is support from the PE teacher which is essential to autonomous motivation towards not just PE, but also leisure-time PA.

In the present study it was hypothesized that support may mediate PA behaviors in various goal setting conditions. Therefore, taking into consideration the relatively healthy status of the examined students, a target of 10,000 steps a day was set as the most appropriate goal for late adolescents, whilst still being challenging. It also served the prospective health and long-term related aims. In the case of younger adolescents and children, the recommended number of steps a day is 12,000 [8] and this was used as an achievable target in our research for these age categories. This level was set to examine whether the setting of predetermined goals by an external source (i.e., a PE teacher) to a group (school class) would bring different results than when the goals were self-set by school age individuals.

## 2. Methods

### 2.1. Participants and Procedure

The study included data collected in 2015 from 65 adolescents (mean age 17.2 ± 0.2), 35 of whom were boys (body mass 71.6 ± 9.5; height 181.4 ± 5.6; BMI 21.5 ± 2.4) and 30 were girls (body mass 57.5 ± 7.8; height 167.0 ± 5.7; BMI 20.5 ± 2.4); 74 young adolescents (mean age 15.3 ± 0.2) 39 of whom were boys (body mass 59.2 ± 9.4; height 173.0 ± 6.7; BMI 19.7 ± 2.4) and 32 were girls (body mass 58.0 ± 7.7; body height 164.3 ± 5.2; BMI 21.5 ± 2.6); and 57 children (mean age 11.5 ± 0.4) of whom 27 were boys (body mass 44.4 ± 10.2; body height 150.3 ± 8.2; BMI 19.4 ± 3.3) and 30 were girls (body mass 42.4 ± 10.2; body height 149.7 ± 8.0; BMI 18.7 ± 2.9). Body mass and height were determined with the use of anthropological instruments.

The participants were recruited from the same grade level of standard urban schools in the city of Poznań, Poland. The sample unit was a school class. Questionnaires were completed in whole-class groups during one regular school lesson in quiet classroom conditions and took approximately 20 min to complete. Physical fitness and anthropological measures were taken during PE classes.

An intervention was designed with two different experimental groups. The groups were selected randomly. Experimental group 1 (“Goal set” group) had a daily goal of 10,000 steps which they were to achieve for 8 consecutive weeks (the level was 12,000 for young adolescents and children). In the same period of time, students from the experimental group 2 (“Do your best” group) were asked to do as many steps they could and wanted every day. Participants in both experimental groups wore a Vivofit^®^ wrist band activity tracker (Garmin, Lenexa, KS, USA) 24 h a day for 8 consecutive weeks. For the “Goal set” group the target was set at the beginning of the intervention and was displayed on the screen as the number of steps to be done to reach the daily goal. For the “Do your best” group no target was set. Participants could also see the number of steps they had taken on the activity trackers’ screens. Each participant had an Internet account created on the Garmin Connect program and could follow their progress and weekly trends. Data was downloaded to the account once a week by a member of the research team.

Pre-test examination took place prior to the introduction of the program, and post-testing was undertaken immediately after cessation of the intervention. All other daily and weekly activities (school PE program, educational and sporting activities) were carried out according to the normal routine for both groups.

### 2.2. Physical Activity

The level of PA was determined with a Physical Activity Screening Measure [30]. This measure corresponds to the average number of days per week with at least 60 min spent undertaking various forms of PA during which, in the participants’ subjective opinion, their heart rate increased and they experienced a feeling of shortness of breath (higher breathing frequency). Participants were asked to answer two questions: P1: Over the past 7 days, on how many days were you physically active for a total of at least 60 min per day? P2: Over a typical or usual week, on how many days are you physically active for a total of at least 60 min per day?

The MVPA index was calculated based on the following formula: MVPA = (P1 + P2)/2 where: MVPA = PA index; P1 = number of physically active days during the past 7 days; P2 = number of physically active days during a typical (usual) week.

### 2.3. Classmate and Teacher Support Measure

In the case of external support, two scales containing five questions each were designed to assess classmate and teacher support during PE lessons. These scales were based upon the Classmate and Teacher Support Scale. Test-retest correlations were 0.69 [31]. Based on the results from 11 to 16 year old girls and boys from seven different countries Torsheim et al. [32] indicated that the Classmate and Teacher Support Scale is well suited for use in large social surveys and can be used in spite of potential language and cultural differences.

In this study it was decided that an amended version of the Classmate and Teacher Support Scale, adjusted to the PE environment, would be used. Two items from the original Teacher Support Scale and three items from the Classmate Support scale were used. In addition, a total of five extra items were additionally designed and adjusted to the PE environment by a panel of experts (psychology, physical activity, physical education). Three extra items were added to assess teacher support during PE, and two to assess classmate support during PE. The internal consistency of the scales had been previously established using Cronbach’s Alpha test in a study on Polish pupils [33]. For the teacher support scale it was α = 0.91, and for classmate support it was α = 0.87.

The statements on the classmate support scale were as follows: (1) other students accept me as I am; (2) most of the students in my class are kind and helpful; (3) I am often picked to play on various teams; (4) I get positive feedback from my peers when I play; and (5) the students in my class enjoy being together. The items on the teacher support scale were: (1) our teacher treats us fairly; (2) when I need extra help, I can get it; (3) I get positive feedback from my teacher when I play; (4) our teacher makes sure we all treat one another fairly; and (5) the teacher lets us express our opinions. Statements 1 and 2 came from the original questionnaires [31] and the other three were developed by the research team. The examined individuals had to assess, on a 5-point Likert scale, whether they agreed (strongly agree) or disagreed (strongly disagree). The total score could amount up to 25 points on each of the scales.

### 2.4. Statistical Analyses

Data analyses were performed using the Statistica software (version 10.0, StatSoft, Krakow, Poland) and statistical significance was set at a probability of *p* < 0.05. Descriptive statistics are presented below as means and standard deviations, and were performed for pre-test and post-test values of MVPA, Peer Support, Teacher Support and Average Steps (post-test only). The correlation in correlation matrix was used to measure the strength of association of MVPA, Classmate Support, Teacher Support and Average Steps (post-test only) as well. Comparisons were calculated for groups and gender categories using a two-way (groups × gender) ANOVA.

### 2.5. Ethics

All participants received a letter describing the purpose and procedure of the study. Written consent was obtained from the parents or guardians of minors. Students were also informed about the anonymous and voluntary nature of their participation, that the study records would be kept confidential, and that their individual contributions would be unidentifiable in the final report. The return ratio was high, with only a very few questionnaires (amounting to just 3%) having to be rejected because of missing data or misleading answers. The study protocol was approved by the Local Bioethics Committee of Karol Marcinkowski University of Medical Sciences in Poznań (decision No. 126/15).

## 3. Results

Table 1 presents mean pre-test and post-test characteristics for the examined variables. 

Table 2 shows the differences between steps (daily average figure) covered by the experimental groups in each age category. Adolescents of both groups took more than 10,000 steps a day (the target for this age category), while young adolescent girls from both of the groups did not meet the target (12,000 steps for this age category). In the group of children, only the girls from the “Do your best” group met the criterion. Generally, in most of the cases the daily average was higher in the “Do your best” group in all categories in adolescent, and young adolescent boys, and in the girls from the children group. A two-way ANOVA showed there were no interactional effects in adolescents. In young adolescents, gender factor (*p* = 0.0038) explained 11% of variance and group factor (*p* = 0.0244) explained 7%. In children, group factor (*p* = 0.0022) explained 9% of variance. 

Table 3 presents the correlation matrix between MVPA, Classmate Support, Teacher Support in pre-test and post-test, and Average Steps among youth. Overall, the MVPA from pre-test was positively associated with all variables except Classmate support from post-test, where no association was found. Classmate Support and Teacher Support from pre-test was not associated with the Average Steps variable. MVPA and Teacher Support from post-test was positively associated with all variables.

A two-way ANOVA showed that there were interactional effects between gender and Teacher Support in both pre-test (*F* = 16.12, *p* < 0.05) and post-test (*F* = 18.21, *p* < 0.05). Overall, boys from both experimental groups (“Goal group” and “Do your best group”) received more support than girls from PE teachers. A two-way ANOVA showed that there were no interactional effects in either pre-test or post-test between other variables when compared to group and gender (group × gender). There was no interactional effect (two-way ANOVA) between gender, group and MVPA either in pre-test or in post-test. A similar situation was observed in the case of Average Steps and with Classmate Support, where a two-way ANOVA also showed no interactional effects between gender and groups in pre-test nor in post-test.

## 4. Discussion

The aim of this study was to investigate the assumed effectiveness of different goal setting conditions and the possible mediating role of support on MVPA. Both tested goal strategies have led to similar outcomes in terms of meeting recommended step levels. Generally, there was a positive link between Teacher Support and gender, where boys from both groups (“Goal group” and “Do your best group”) received more support than girls from PE teachers. Unfortunately, no interactional effects in either pre-test or post-test between other variables were observed when compared to group and gender (group × gender). The goal (predetermined and “do your best”) did not mediate differences in either MVPA or number of steps.

Our data indicated that there were significant positive correlations (*p* < 0.05) between MVPA and Teacher Support in post-test with all variables. Interestingly, there was no such relationship between MVPA (pre-test) and Classmate Support (post-test), Classmate Support (pre-test) and Average Steps and Teacher Support (pre-test), and Average Steps (insignificant difference). It appears that by supporting students during the PE process, PE teachers play an important role in determining the MVPA levels and also influence after school PA levels. Hagger et al. [26] assumed that an increase in support from PE teachers, parents and classmates would also increase the motivation to undertake PA in during leisure time. In the present study, this hypothesis was confirmed only in relation to PE teachers. However, we also found a correlation between MVPA (post-test) and Classmate Support (post-test), which indicates the importance of Classmate Support. The phenomenon of Classmate Support as a strong mediator of PA of youth is also reported in the study of Sallis et al. [34]. Likewise, Duncan et al. [35] also noticed that classmate support is an important factor, even stronger than family support.

In our study, the lack of changes in MVPA over the 8 weeks, despite the goal strategy, might have been caused by few factors. It is possible that the reasonably high level of MVPA at the baseline of the intervention did not flag any need for its increase and that as a consequence achieving the daily target of steps was a reachable target for the students in both experimental groups. It is also possible that the intervention did not bring significant changes in MVPA behaviors because students were not given the chance to choose a strategy to achieve a given goal, as the strategy was assigned to randomly selected classes rather than individuals. According to Locke and Latham [16] the relationship between group goals and individual goals influences group performance. The authors claim that when goals are compatible there is a positive effect, but when goals are incompatible the effects can be detrimental to the group’s performance. They found a positive correlation exists between sharing information within the group and group performance. In the case of group goals, feedback needs to be related to the group rather than individuals in order for it to improve the group’s performance. We are also aware that the level of commitment to achieving goals might have differed among students, but since the intervention was undertaken in a school setting, it is possible that all, or many of the students (from both groups) might have treated the goals as a school homework task. Likewise, it is possible that this might be one of the reasons why the results of the present study did not show any significant variations between groups even with different goal strategies.

Chatzisarantis and Hagger [36] found that autonomous motivation and intentions mediate changes to PA behaviors, which are enhanced by an autonomy-supportive environment, rationale, and feedback. In the case of our study, the students’ only feedback was the number of steps displayed on an activity tracker. Research in sport [37] shows that in the case of motor behavior, consistently demonstrated feedback (sometimes referred to as knowledge or results of knowledge of performance) has a motivating effect, regardless of whether it is tied to specific goals or not. Additionally, research by Kyllo and Landers [38] revealed that performance goal setting in sport and exercise domains is effective at improving performance if the goals are set specifically, in both the short and long term, by individuals themselves, and if they are made public to others. In our study, having continuous feedback of one’s performance (number of daily steps done) might have been a motivating factor in itself, despite the goal and it is also possible that students from the “Do your best” group set their own goals (i.e., according to health recommendations). The above factors could also play a role in generating similar results between the groups; students nowadays can easily check health-related recommendations via the Internet. It is possible that students from the “Do your best” group might have checked the number of steps required to meet health recommendations and subsequently followed the guidelines. Cullen et al. [13] point out that keeping daily records and receiving feedback are likely to enhance motivation to complete actions that are relevant to goal accomplishment and that, in turn, this motivates people to initiate further goal setting. On the other hand, failure to achieve a predetermined goal could have a negative impact on self-efficacy. That said, the same applies to self-set goals in a “do your best” strategy. When goals are set specifically, achievement can be measured, thus enhancing the likelihood of attainment and the potential for an increase in self-efficacy. Goals can thus be set higher and made more challenging so that they lead to higher performance [12]. Nevertheless the PE environment (with different education objectives than sport) needs to be explored in more depth in this context in further studies. This issue needs to be investigated separately due to the fact that self-monitoring and feedback at the individual’s convenience could be used as effective educational tools in the school setting, especially with girls or overweight and obese children. 

With regards to support, group was not a factor differentiating the level of support. The factor of PE teacher support was noticed to be the most influential one in our study in both groups. Intervention did not change much in the level of teacher support when compared to pre-test levels. Cox and Ulrich-French [39] found that positive relationships with both teachers and peers are associated with optimal PE experience and may afford some advantages with even relatively small support. However, interestingly, Hagger et al. [40] found that a model of perceived autonomy support in PE predicted autonomous motivation, intentions and behavior in a leisure-time PA context with students from Britain, Greece, Singapore, but had to be rejected with a sample of Polish adolescent students. Perhaps with Polish students, more sophisticated models and methods need to be employed, or the level of support is already reasonably high. However, it needs to be mentioned that changing support levels was not the direct aim of the intervention in our study, as no manipulation of either teacher or classmate support was included. Nevertheless, the findings of the study suggest that facilitating students (across the age categories) to take up leisure-time PA may begin in school PE with teachers and the support they provide. According to the findings of a study by Jackson-Kersey and Spray [41], if students perceived their PE teacher to provide inadequate support for their basic psychological needs, PE tasks became less appealing over time. When PE teachers inadequately supported autonomy, competence, and relatedness, students evoked feelings of boredom and disinterest. However, Lim and Wang [42] found that despite lacking motivation in PE classes, students may still be active outside school, participating in PA chosen based on their efficacy and with affective appraisal by significant others.

The findings from our study could imply that setting goals may be an insufficient way of enhancing youth health-related behaviors, specifically when the initial level of MVPA is already moderate. It is probably worth mentioning that students from both groups had the same school routine (four PE 45-min lessons scheduled each week). PE lessons might have added a reasonable number of steps to a daily goal, but this applied to students from both groups and there were no major differences between weekly steps in either of the groups. The study also revealed that older girls (late adolescents) are more vulnerable in terms of support received from PE teachers and that their level of MVPA is relatively lower than their younger peers, especially boys. Changing this would require elevating the level of support, specifically when PE teachers are concerned. Earlier work [43] has indicated a significant increase in low active adolescent girls when receiving high support from a PE teacher. In a longitudinal study of inactive adolescent girls [44], perceived support from PE teachers, peers, and parents was also found to be one of the strongest and most consistent predictors of changes in PA. Barr-Anderson et al. [45] highlighted the importance of the impact of teachers’ attitudes and expectations, especially in PE, and the importance of creating an environment that supports gender equality and builds confidence in girls’ abilities. Furthermore, it has to be taken into consideration that influences on girls’ PA are multifactorial and include a variety of psychological, social, and environmental correlates [34].

Autonomy-supportive environments (especially that provided by teachers) have been proven to be effective in other studies [36,46], but it also has been shown that such environments only promote the performance of health-related behaviors when they facilitate an autonomous motivational style [47]. The findings of a study by Vensteenkiste et al. [48] indicated that it is not just the perception of support, but also the type of goals people pursue (termed “intrinsic” and “extrinsic”) that matters. The effect on PA behavior (specifically sport participation) is greater when autonomy support is combined with intrinsic goals. Nothwehr and Yang [49] found that self-directed goal setting tends to be connected with positive behavioral strategies. This is more advantageous for goals set by individuals themselves, as well as for focused, rather than general goal setting. This is certainly possible with individuals aware of their needs and focused on health or performance improvement, but the question of whether this can be utilized in a school (class) PE setting is still moot.

The experimental design of the study is its strength, but to fully take advantage of the benefits of activity trackers, step-based interventions and large, randomized controlled trials are required. The limitations of this study concern the relatively small sample size and the good baseline MVPA level, which made achievement of the target daily goals relatively easy. Furthermore, the predetermined setting of the goals, which were not personally selected, might have been an issue. Feedback on the number of steps in each group might also have been an issue. In future studies it would perhaps be better if the “Do your best” group had their monitors blinded to enable them to perform to their capabilities rather than the potential target. The technological novelty of the activity trackers may have played a motivational role, but participants might have fulfilled the goals by virtue of knowing that they were being monitored. They could also have checked the recommended criteria on the Internet and followed them accordingly. This would be an educational success if this was proven true, but unfortunately no measures of such behaviors were taken. In further studies this needs to be considered as an important and potentially mediating factor. Interventions help to create a more in-depth picture of how goal setting strategy may impact youth health and it would also be of value in future studies for the factor of an autonomy supportive environment to be added and the long-term effect of achieving the goals (recommended PA doses) to be evaluated.

## 5. Conclusions

The present study extends the current knowledge about the effectiveness of various goal strategies in youth. Results suggest that both strategies seem to be similarly effective, specifically when feedback provision is available to each participant. Classmate Support in PE was reported to be reasonably high, but the findings show that it does not, as such, play a significant role in increasing MVPA behaviors of youth. The significantly lower support of PE teachers afforded to adolescent girls should be embedded into the teaching context of PE students and counteracted in school setting realities. Teachers can use individualized counseling time and awareness talks (this can be also done in a group setting), self-paced challenges with self-monitoring task cards or behavioral contracts. Planning calendars can also be utilized and when adopting modern technology (activity trackers, heart rate monitors, mobile phones applications), Internet-based programs could be adopted at the person’s convenience. The lack of mediating effect of social support from classmates on MVPA should also be addressed with changes in school setting, specifically in PE curricula.

## Figures and Tables

**Table 1:** Means and standard deviations in examined variables in pretest and posttest.

Variables	Goal Group	Do Your Best Group
	M ± SD	M ± SD
MVPA (pretest)	4.2 ± 1.46	4.3 ± 1.25
MVPA (posttest)	4.3 ± 1.59	4.1 ± 1.54
Classmate support (pretest)	20.8 ± 4.31	20.0 ± 4.39
Classmate support (posttest)	19.9 ± 3.71	20.4 ± 4.08
Teacher support (pretest)	21.4 ± 3.55	20.3 ± 4.44
Teacher support (posttest)	19.9 ± 4.45	19.4 ± 4.72
Average steps (posttest)	10,761.6 ± 2830.6	11,954.5 ± 3498.9

**Table 2:** Means, standard deviations and differences in daily steps across age categories.

Gender	Goal Group (Steps av/8 Weeks)	Do Your Best Group (Steps av/8 Weeks)	Source	ANOVA (Gender × Group)		
Adolescents (age 17.5)				*F*	*p*	eta^2^
Boys	10.015 ± 2.517	12. 26 ± 2.515	Gender	0.0	0.9709	0.00
Girls	11.112 ± 2.950	10.985 ± 3.176	Group	1.51	0.2235	0.02
			Gender × group	1.99	0.1680	0.03
Young adolescents (age 15.5)						
Boys	12.374 ± 2.955	14.360 ± 5.304	Gender	8.960	**0.0038**	0.11
Girls	10.094 ± 1.083	11.817 ± 2.229	Group	0.026	**0.0244**	0.07
			Gender × group	5.298	0.8705	0.00
Children (age 11.5)						
Boys	10.039 ± 2.949	11.816 ± 3.613	Gender	0.003	0.0963	0.00
Girls	9.581 ± 4.420	12.172 ± 4.403	Group	5.560	**0.0022**	0.09
			Gender × group	0.193	0.0662	0.00

Note: Significant differences are indicated in bold.

**Table 3:** Correlation matrix between MVPA, Classmate support, Teacher support in pretest and posttest and Average steps in posttest.

Variables	MVPA (Pretest)	Classmate Support (Pretest)	Teacher Support (Pretest)	MVPA (Posttest)	Classmate Support (Posttest)	Teacher Support (Posttest)	Average Steps (Posttest)
MVPA (pretest)	-	**0.14**	**0.23**	**0.45**	**0.09**	**0.24**	**0.19**
Classmate support (pretest)	**0.14**	-	**0.24**	**0.16**	**0.43**	**0.20**	0.12
Teacher support (pretest)	**0.23**	**0.24**	-	**0.18**	0.17	**0.50**	−0.07
MVPA (posttest)	**0.45**	**0.16**	**0.18**	-	**0.24**	**0.26**	**0.18**
Classmate support (posttest)	0.09	**0.43**	**0.17**	**0.24**	-	**0.38**	**0.18**
Teacher support (posttest)	**0.24**	**0.20**	**0.50**	**0.26**	**0.38**	-	**0.18**
Average steps (posttest)	**0.19**	0.12	−0.07	**0.18**	**0.18**	**0.18**	-

Note: Significant correlations are indicated in bold.
